# The Short and Brave Life of Gaetano Perusini: A Tribute to His Role in Shaping Neuroscience

**DOI:** 10.7759/cureus.54240

**Published:** 2024-02-15

**Authors:** Francesco Pellegrini, Musa Mutali, Marco Zeppieri

**Affiliations:** 1 Ophthalmology, Santo Spirito Hospital, Pescara, ITA; 2 Optometry, University of Benin, Benin, NGA; 3 Ophthalmology, Africa Eye Laser Centre, Benin, NGA; 4 Ophthalmology, University of Udine, Udine, ITA

**Keywords:** memory loss, udine, alzheimer's disease, neuroscience, perusini

## Abstract

Gaetano Perusini was an early enigma in neuroscience. In an age where myths and religion still held tightly to medical knowledge, Dr. Perusini was a trailblazer, inventor, and decorated forerunner. Born in Udine, worshiped in Italy, educated across Europe, published all over the western hemisphere, and taken away from us during a time of worldwide strife, his story continues to fascinate us today. This is a short chronicle of the major events in his life that also celebrates his widely acclaimed influence on understanding Alzheimer’s disease.

## Editorial

The purpose of this editorial is to rekindle the scientific community's interest in Dr. Gaetano Perusini, who, in a short time, managed to make a substantial contribution to Alzheimer’s disease. His research in Italy was fundamental in giving rise to the specific characterization of many neuropathological aspects of this pathology, which in the 1970s was defined as Alzheimer-Perusini’s disease. His impressive results were later confirmed in the studies that followed. The premature death of the neuroscientist during the First World War certainly prevented new ingenious research in the field of neuroscience. Despite his young age, Dr. Perusini managed to make a fundamental contribution to neuropathology, even if he was not easily accepted and approved by the scientific community in those times.

Almost everything we know today about Gaetano Perusini comes from the memories of his inseparable friend and colleague, Dr. Ugo Cerletti, another important name in the history of neurology and psychiatry. Prof. Bruno Lucci has also written several books that provide interesting details about this gifted neuroscientist and his fundamental historical research [1]. The aim of this short biography is mainly based on these two books unless otherwise noted in the bibliography.

Gaetano Perusini’s ancestry dates back to the 14th century in Northern Italy. He was born in Udine, Italy, on February 24, 1879, to Dr. Andrea Perusini and Paolina Cumano. That was a particularly difficult period for Italy, considering that the nation was united in 1861 and was involved in World War I (WWI) in 1915. Andrea Perusini was a physician and the director of the Udine City Hospital for 20 years (March 1866 to April 1886). He had a fundamental role in organizing the sanitary facilities for the mentally ill in Udine; this attracted knighthood status from the Republic of Italy.

On the maternal side, the Cumano family was of Venetian origin. Paolina married Andrea Perusini, while her sister married Andrea’s brother. Gaetano Perusini was raised in a family with strong moral principles, intellectual prowess, honesty, willpower, a sense of sacrifice, and patriotism. His family was considerably wealthy (before WWI), which allowed him to enjoy an internationally flavored cultural experience and to study without the worries of financial difficulties. The young Perusini lost his father when he was only seven, and this forced his mother, Paolina, to take charge of her three sons with the assistance of a German teacher.

Perusini completed his classical studies at the Royal High School in Udine. He obtained his high school diploma at age 16. Along with his brother Giacomo, he applied to the University of Pisa to study medicine and agronomy. By the end of 1899, Perusini transferred to the University of Rome to attend the Hospital Clinic directed by the famous Prof. Giovanni Mingazzini. While in Rome, he met Ugo Cerletti, at the time a medical student as well, who, like him, would later go on to make history. It is thanks to Cerletti that we now know quite a bit about Gaetano Perusini, especially considering that the Perusini family archives, photos, and personal belongings were destroyed when the Austro-Hungarian army invaded Friuli and Veneto (Northern Italy) during WWI. Dr. Cerletti defined Perusini as “well-mannered, impeccably dressed” with an “aristocratic look” who, however, was a truly ”hard worker” who “toned down his lifestyle to reflect a Franciscan severity and simplicity” [1].

Cerletti and Perusini spent a lot of time together. They both graduated in 1901 from the University of Rome. Perusini's dissertation thesis in psychiatry entitled *L’apparecchio Passivo Di Masticazione Nei Delinquent* (The Passive Apparatus of Chewing in Criminals) was later published. His research was based on analysis from an anthropometric perspective regarding the oral cavity of 200 male criminals convicted of murder. The thesis, which today might appear bizarre, was part of the heated scientific debate concerning the anthropometric differences between criminals compared to 'healthy' subjects. Immediately after obtaining his medical degree, Perusini began to assiduously attend the departments directed by two professors who left indelible traces in the fields of neuroanatomy and neuropathology, mainly Prof. Primo Dorello [2] and Prof. Giovanni Mingazzini [3].

Perusini's friendship with Cerletti extended beyond university life and continued even during the summer periods. Between 1903 and 1905, they took on a research project together to study families affected by cretinism (endemic hypothyroidism) in Valtellina (Lombardy). Contrary to the common belief of those times, the two young doctors were convinced that the endemic goiter could not be explained by Morel's "degenerative theory" (also accepted by Kraepelin), but that it was linked to external "environmental" causes. This research was then considered fundamental, especially in light of the fact that, after a few years, the Italian Permanent Committee for the Fight against Cretinism adopted the guidelines for adding iodine to cooking salt [4].

Perusini's research and discoveries represent a starting point for professional growth as they push his meticulous mind forward in the search for details and truth against unproven theories and imposed preconceptions. Perhaps one of the most important aspects of this great scientist is that, despite his brief life, he represented a shining example of dedication to medical science and others, with a tireless sense of persistence, courage, and altruism. It is no coincidence that Cerletti wrote that Perusini "always wanted to keep the good from evil separated".

In addition to the clinical studies he performed, Perusini always supported his research with laboratory studies. He had the opportunity to attend the prestigious Departments of Histopathology and Anatomy in Munich between 1904 and 1905, with Dr. Schmaus and Dr. Alzhemier, and the one in Zurich in 1906 with Prof. Von Monakow. In 1907, at the age of only 28, he was appointed clinical assistant at the Psychiatric Clinic in Rome, directed by Prof. Sciamanna. Before the age of 30, Perusini was well respected and esteemed by colleagues on an international level. He was invited to be on the editorial board of *Folia Neurobiologica*, a Dutch scientific journal founded by Hemka. Three years later, in 1910, he obtained a teaching position in the Clinic for Neurological and Mental Diseases, in which the examination committee declared that "Dr. Perusini had uncommon scientific merits.”

In 1913, he was hired as an assistant at the Psychiatric Hospital of Mombello (Milan). Two years later, when Italy entered the war against Austria-Hungary, Perusini volunteered for military service, while hiding his academic status, to fight on the battlefield. After his death, his commander reported that Perusini was an "energetic and tireless worker and always offered his assistance all the time and on any occasion." Moreover, he stated that “he was always modest and always chose the humblest and most difficult tasks." On more than one occasion, Perusini gave the army permission to use his home in Udine and the family properties in Friuli Venezia Giulia.

In November 1915, he was assigned to help the sick at San Floriano (in the province of Gorizia). On the morning of November 28 that same year, the clinic was hit by Austrian shrapnel, despite conventions already in force at the time forbidding the bombing of field hospitals. The ballistic trauma caused by the explosions caused penetrating wounds to his lungs and a broken leg. He immediately understood the seriousness of the situation. Those who rescued him reported that he said, "It's over for me." Shortly before he died, he received a visit from two officers personally sent by the King of Italy. His friend Cerletti remained by his side throughout his sickness and assisted him till his death on December 8, 1915, at 5 p.m., near Cormons (in the province of Gorizia).

He was later awarded the Silver Medal for military valor. There were numerous commemorations to express the recognition, support, and pain of fellow citizens and the entire academic world. Dr. Ely Jelliffe, Chief Editor of the American Journal of Nervous and Mental Disease, wrote that "no more striking instance of the irreparable tragedy of the European war presents itself than in the untimely death of Gaetano Perusini." Perusini's studies were published widely in the journals of various countries, through which he became better known for his work than even in his land. However, the field of neurology and psychiatry has suffered the loss of an earnest and brilliant worker whose future held much promise.

Perusini wrote about 50 scientific papers, the first of which were published when he was only 23 years old. The publications were in various fields of medicine, which ranged from cretinism to polymastia, from dentistry to neurology, and of course, pathological anatomy, especially regarding the nervous system. In this latter field, Perusini left an indelible mark; his discoveries and annotations have stood the test of time until now. A more detailed description of his research on Alzheimer's disease can be found elsewhere [4]. However, it is important to mention a few here: (1) the concurrence of neurofibril changes with plaque formation; (2) observations on the nature and origin of senile plaques; (3) the understanding of the progressive process of dementia that probably begins years before the clinical manifestation (an old pathological process that advances over the course of the years); (4) the important changes in glial cells over time; (5) the discovery of a compound now known as β-amyloid (to which he said, “My hypothesis is that a kind of cement glued the fibrils together”); and (6) the correlation between the blood vessel changes and the destruction of neurons.

Alzheimer-Perusini’s disease was a commonly used eponym of the disorder until 1978, when Prof. Katzman changed the classification and terminology of dementia, thus distancing Perusini’s name from this pathology. In 1911, Dr. Alzheimer showed high esteem and recognition when he stated, "Perusini believes that these cases characterize a distinct disease, partly for clinical and partly for histological reasons" [5]. In conclusion, Alzheimer’s disease should probably be best renamed 'Alzheimer-Perusini’s disease', thus immortalizing the two neuroscientists who both equally contributed to the discovery and characterization of this form of dementia.
